# Assessment of antibody levels to SARS-CoV-2 in patients with idiopathic inflammatory myopathies receiving treatment with intravenous immunoglobulin

**DOI:** 10.1007/s00296-023-05350-1

**Published:** 2023-06-27

**Authors:** Sangmee Sharon Bae, Emmanuelle Faure-Kumar, Kathie Ferbas, Jennifer Wang, Ani Shahbazian, Linh Truong, Howard Yang, Maureen McMahon, John D. FitzGerald, Christina Charles-Schoeman

**Affiliations:** 1 David Geffen School of Medicine, Division of Rheumatology, University of California Los Angeles, Los Angeles, CA USA; 2 David Geffen School of Medicine, Vatche and Tamar Manoukian Division of Digestive Diseases, University of California Los Angeles, Los Angeles, CA USA; 3 David Geffen School of Medicine, Department of Pediatrics, University of California Los Angeles, Los Angeles, CA USA

**Keywords:** Idiopathic inflammatory myopathies, Dermatomyositis, Polymyositis, Intravenous immunoglobulin, Anti-SARS-CoV2 antibodies

## Abstract

Antibodies to Severe Acute Respiratory Syndrome-Coronavirus 2 (SARS-CoV-2) have been reported in pooled healthy donor plasma and intravenous immunoglobulin products (IVIG). It is not known whether administration of IVIG increases circulating anti-SARS-CoV-2 antibodies (COVID ab) in IVIG recipients. COVID ab against the receptor binding domain of the spike protein were analyzed using a chemiluminescent microparticle immunoassay in patients with idiopathic inflammatory myopathies (IIM) both receiving and not receiving IVIG (IVIG and non-IVIG group, respectively). No significant differences in COVID ab levels were noted between IVIG and non-IVIG groups (417 [67–1342] AU/mL in IVIG vs 5086 [43–40,442] AU/mL in non-IVIG, *p* = 0.11). In linear regression models including all post-vaccination patient samples, higher number of vaccine doses was strongly associated with higher COVID ab levels (2.85 [1.21, 4.48] log AU/mL, regression coefficient $\beta$ [95% CI], *p* = 0.001), while use of RTX was associated with lower ab levels (2.73 [− 4.53, − 0.93] log AU/mL, $\beta$[95%CI], *p* = 0.004). In the IVIG group, higher total monthly doses of IVIG were associated with slightly higher COVID ab levels (0.02 [0.002–0.05] log AU/mL, *p* = 0.04). While patients on IVIG did not have higher COVID ab levels compared to the non-IVIG group, higher monthly doses of IVIG were associated with higher circulating levels of COVID ab in patients receiving IVIG, particularly in patients concomitantly receiving RTX. Our findings suggest that IIM patients, especially those at increased risk of COVID infection and worse COVID outcomes due to RTX therapy may have protective benefits when on concurrent IVIG treatment.

## Introduction

Increasing numbers of the general population have been infected with severe acute respiratory syndrome coronavirus 2 (SARS-CoV-2; COVID-19), and the development as well as distribution of vaccines has occurred at an unprecedented pace [1]. With high rates of infection and vaccinations of the general population, healthy donor plasma pooled products such as intravenous immunoglobulins (IVIG) now contain antibodies to COVID-19. Romero et al. demonstrated that SARS-CoV-2 antibodies increased by 10–50 times in plasma pools and IVIG products from May 2020 to September 2021, mirroring the exposure of COVID-19 in the population [2]. Neutralization studies with wild-type SARS-CoV-2 virus showed that IVIG products had neutralization potency, which raises the question of whether IVIG offers protective or therapeutic benefit in the ongoing pandemic [2].

IVIG has immune modulatory properties that historically have demonstrated effectiveness in serious viral infections such as influenza, severe acute respiratory syndrome (SARS), and Middle East respiratory syndrome (MERS) with passive transfer of both virus specific and cross-reactive antibodies as a proposed mechanism for its efficacy [3–6]. A systematic review of 6 randomized control trials investigating the effectiveness of IVIG being used to treat hospitalized patients with COVID-19 demonstrated that IVIG does not improve clinical outcomes including mechanical ventilation, intensive care unit admission or mortality [7]. However, whether high dose, regular infusions of IVIG in the outpatient setting used for other indications such as inflammatory myositis offer protective benefits in its recipients remains unknown. If infusion of plasma products can provide at least short-term benefit as seen in data from convalescent plasma [8], products such as IVIG which are infused regularly on a weekly, bi-weekly or monthly basis may be more intriguing, especially in patients with underlying autoimmune diseases who are at high risk for severe COVID-19 infections. No work to date has reported whether regular infusions of high-dose IVIG in the outpatient setting increase circulating levels of SARS-CoV-2 antibodies.

Idiopathic inflammatory myopathies (IIM) are a group of systemic autoimmune diseases characterized by inflammation of the skeletal muscle. IVIG has been widely used as a therapy for IIM and was recently approved by the U.S. Food and Drug Administration (FDA) for the treatment of the dermatomyositis(DM) subtype and is typically administered in high doses (2 g/kg) each month. The current work evaluates SARS-CoV-2 antibody (COVID ab) levels in a single-center cohort of IIM patients comparing COVID ab levels in patients receiving regular IVIG infusions to patients not receiving IVIG.

## Patients and methods

### Study population

Biospecimens from the UCLA myositis cohort were used for the analysis. The UCLA myositis cohort is a longitudinal cohort that consists of 350 patients (68% DM, 20% polymyositis, 5% inclusion body myositis, 7% other). All patients met EULAR/ACR Classification Criteria for adult IIM for at least ‘probable’ IIM [9]. All plasma samples collected between March 2021 (after vaccination for the general public available in the USA) and February 2022 were analyzed, which were a total of 45 samples. All subjects gave written informed consent for the study approved by the Human Research Subject Protection Committee at UCLA (UCLA IRB# 10-001833).

All post-vaccination specimens were collected after patients had received at least one COVID messenger RNA(mRNA) vaccine (BioNTech, Pfizer vaccine BNT162b2 or Moderna vaccine mRNA-1273). Patients receiving regular IVIG treatment (IVIG group) were compared to patients who did not receive IVIG for at least 3 months prior to blood draw (non-IVIG group). Pre-vaccination stored samples between 2010 and March 2021 were also analyzed. COVID infection was assessed before the blood draw and also after blood draw until May 2022 prior to the spread of the omicron variant.

### SARS-CoV-2 antibody testing (COVID ab)

Antibody testing was performed using a chemiluminescent microparticle immunoassay to detect IgG antibodies against the receptor binding domain of the Spike protein (anti-RBD) per the manufacturer’s instructions and previously published protocols [10]. Positive anti-Spike IgG titers were defined as equal to or more than 50 arbitrary units per milliliter (AU/mL). Negative titers were < 50 AU/mL.

### Statistical analysis

COVID ab levels, as well as demographic and clinical characteristics, were compared between the IVIG and non-IVIG group using Student’s *t*-test for normally distributed data, the Wilcoxon test for skewed data, or the chi-square test for categorical data.

Univariate and multivariate linear regression analyses were used to evaluate predictors of COVID ab titers. COVID ab titers were log-transformed and included as the outcome in linear models. COVID vaccine predictors (number of vaccinations, time from vaccination to blood sample collection, type of vaccine [Pfizer vs Moderna], and medication use) were analyzed in univariate linear regression analysis of all post-vaccination samples. IVIG-related predictors (IVIG dose, time from last IVIG to sample collection, IVIG administration schedule [weekly or biweekly vs monthly]) were analyzed in univariate analysis of the IVIG group. Multivariate linear regression analyses included clinical variables known to be associated with COVID ab levels and those significant in univariate analyses.

## Results

### COVID ab levels compared between IVIG and non-IVIG group

We analyzed 24 samples from IIM patients that were receiving regular IVIG treatment (IVIG group) and 21 IIM patients not receiving IVIG (non-IVIG group) with a similar age, sex, and ethnicity distribution (Table 1). Patients were 71% female, 59% white, 20% Hispanic with mean age of 53 years. Most patients (73%) had DM, 42% with interstitial lung disease and antisynthetase ab (22%) were the most common myositis specific autoantibodies. IVIG group patients were receiving 1.2 (0.8–1.7) g/kg, median (IQR) of IVIG every 4 weeks on various schedules (monthly, every 2 weeks, weekly). Patients in the IVIG and non-IVIG groups had similar clinical characteristics, with the exception of medications: use of mycophenolate was higher, while azathioprine and hydroxychloroquine were lower in the IVIG group. The IVIG group had a numerically higher proportion of rituximab (RTX) use compared to the non-IVIG group, which was not statistically significant (50% vs 24%, *p* = 0.08). One patient in the non-IVIG group had COVID infection 13 months prior to blood draw. No other patients had COVID infection prior to their blood draw.

**Table 1:** COVID antibody levels and clinical characteristics of IIM patients receiving regular treatments of IVIG vs matched control non-IVIG IIM group

	IVIG group (*n* = 24)	Non-IVIG group (*n* = 21)
COVID ab levels (AU/mL)^†^	417 (67–1342)	5086 (43–40,442)
Age	52 $\pm$ 10	53 $\pm$ 14
Sex, Female	19 (79)	13 (62)
Ethnicity, Hispanic	6 (25)	3 (15)
Race, White	11 (46)	15 (71)
Black	2 (8)	2 (10)
Asian	8 (33)	3 (14)
Vaccine, Moderna	13 (54)	13 (62)
Pfizer	11 (46)	8 (38)
Time from last vaccination to blood draw, days	102 (21–159)	75 (32–145)
Number of COVID vaccine doses		
1	4 (17)	1 (5)
2	18 (75)	15 (71)
3	2 (8)	5 (24)
COVID infection prior to blood draw	0	1 (5)
Dermatomyositis	19 (79)	14 (67)
Polymyositis	5 (21)	7 (33)
Immune mediated necrotizing myopathy	6 (25)	4 (19)
Myositis autoantibodies		
Antisynthetase	6 (25)	4 (19)
MDA5	6 (25)	2 (10)
SRP	3 (13)	1 (5)
HMGCR	2 (8)	1 (5)
Mi2	0	1 (5)
TIF1 gamma	2 (8)	3 (14)
NXP2	1 (4)	1 (5)
Ro	0	1 (5)
PM-scl	0	1 (5)
Unidentified	2 (8)	3 (14)
None	2 (8)	0
Interstitial lung disease	12 (48)	7 (35)
Medications		
Mycophenolate	17 (71)*	7 (33)
Rituximab	12 (50)	5 (24)
Cyclophosphamide	2 (8)	0
Azathioprine	0*	3 (14)
Methotrexate	3 (13)	2 (10)
Hydroxychloroquine	0 *	3 (15)
TNF inhibitor	1 (4)	0
Prednisone	18 (75)	10 (48)
Prednisone dose (mg/day)	12 $\pm$ 14	7 $\pm$ 14

Values are reported as mean $\pm$ SD or *n* (%) or median (IQR)

^*^p < 0.05 by Student’s *t*-test for normally distributed data (values reported as mean SD), Wilcoxon test for skewed data (values reported as median(IQR)) or chi-square test for count data

**Fig. 1:**
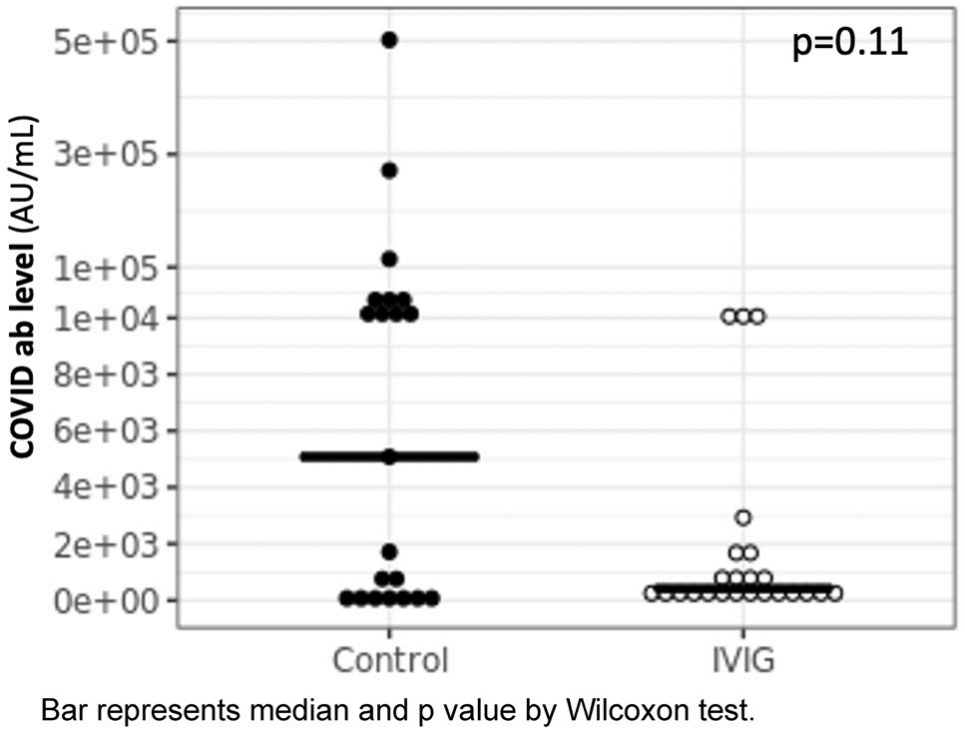
COVID ab levels in patients with IIM who receive regular treatments with IVIG (*n* = 24) control IIM group not receiving IVIG (*n* = 21). Bar represents median and *p* value by Wilcoxon test

COVID ab levels were not significantly different between IVIG and non-IVIG groups with a trend for lower COVID ab levels in the IVIG group (Table 1, Fig. 1). In univariate linear regression analyses, higher number of vaccine doses was strongly associated with higher COVID ab levels (2.85 [1.21, 4.48] log AU/mL, $\beta$[95%CI], *p* = 0.001), while use of RTX was associated with lower ab levels (−2.73 [−4.53, −0.93] log AU/mL, $\beta$[95%CI], *p* = 0.004). None of the other medications were associated with COVID ab levels in univariate models. Time from last vaccination to blood draw and vaccine type (Moderna vs Pfizer) were not associated with COVID ab levels (*p* = 0.11 and 0.35, respectively). In multivariate linear regression analysis, number of vaccine doses and RTX use remained significant predictors of COVID ab titers, while use of IVIG was not a predictor of COVID ab titers (Table 2).

**Table 2:** Multivariate linear regression analysis of predictors of COVID ab levels in IIM patients

	Regression coefficient (95% CI)	*p* Value
IVIG yes (vs no)	−0.98 [−2.75, 0.79]	0.27
Time from last vaccination to blood draw, days	0.008 [−0.003,0.02]	0.16
Number of COVID vaccine doses	**2.50 [0.96, 4.05]**	**0.002**
MMF yes (vs no)	0.96 [−0.67, 2.58]	0.24
RTX yes (vs no)	**−2.40 [−4.18, −0.61]**	**0.01**

Bold values represent variables with *p* value < 0.05

COVID ab titers were log-transformed in linear regression

Analysis includes all post-vaccination samples (*n* = 45)

Azathioprine and hydroxychloroquine were not included in multivariate model due to small numbers leading to model instability

### COVID ab levels in IVIG group

To assess predictors of COVID ab levels in patients receiving IVIG, linear regression analyses of COVID ab levels were performed in the IVIG group (*n* = 24; Table 3). Each variable was adjusted for number of COVID vaccine doses. Higher total monthly doses of IVIG were associated with higher COVID ab levels (*p* = 0.04). Time duration between the last IVIG dose and specimen collection, dose of IVIG at last infusion, or schedule of IVIG treatments (monthly infusions vs doses divided into weekly or bi-weekly infusions) were not associated with COVID ab levels (Table 3).

**Table 3:** Bivariate linear regression analysis of predictors of COVID ab levels in patients receiving IVIG adjusted for number of COVID vaccine doses (*n* = 24)

	Total IVIG group (*n* = 24)		RTX yes (*n* = 12)		RTX no (*n* = 12)	
	$\beta$ (95% CI)	*p*	$\beta$ (95% CI)	*p*	$\beta$ (95% CI)	*p*
IVIG grams at last infusion prior to blood draw	0.06 (−0.04, 0.17)	0.20	**0.11 (0.04, 0.18)**	**0.01**	−0.14 (−0.42, 0.14)	0.25
IVIG grams during last month prior to blood draw (monthly dose)	**0.02 (0.002, 0.05)**	**0.04**	**0.03 (0.01, 0.05)**	**0.01**	2.33 (−2.36, 7.02)	0.29
Days between last IVIG and blood draw, days	0.003 (−0.08, 0.09)	0.95	−0.01 (−0.11, 0.10)	0.86	0.07 (−0.07, 0.22)	0.31
IVIG schedule, weekly or biweekly (vs monthly)	−1.09 (−2.87, 0.69)	0.22	−1.40 (−3.39,0.59)	0.14	−1.89 (−5.25, 1.47)	0.23

Bold values represent variables with *p* value < 0.05

COVID ab titers were log-transformed in linear regression

Each predictor variable in the table adjusted for number of COVID vaccine doses at time of blood draw to assess association with log COVID ab levels

### COVID ab levels in patients on/off RTX

We also performed a stratified analysis by RTX use given the strong association between COVID ab levels and RTX. Among patients who were not on RTX in the past 6 months (*n* = 28), COVID ab levels were not significantly different between IVIG (*n* = 12) and non-IVIG groups (*n* = 16) (1183 [129–8592] vs 13,032 [757–43,778] AU/mL, median [IQR], respectively, *p* = 0.11). Higher number of vaccinations remained associated with higher COVID ab levels in this group (2.79 [1.11–4.47], log AU/mL, $\beta$[95%CI], *p* = 0.002), while time from last vaccination to blood draw and vaccine type (Pfizer vs Moderna) were not associated with COVID ab levels (*p* = 0.68, *p* = 0.72, respectively).

Among patients receiving RTX, there was a trend for higher COVID ab levels in IVIG patients (*n* = 12, 124 [61–481] AU/mL) compared to non-IVIG patients (*n* = 5, 3 [1–21] AU/mL, *p* = 0.15). Higher IVIG dose was significantly associated with higher COVID ab levels in patients receiving RTX (Table 3).

We also performed a two-way ANOVA to analyze the association of RTX and IVIG as independent factors with COVID ab levels and found only RTX, but not IVIG nor the interaction were statistically significant (Table 4).

**Table 4:** Associations of COVID ab levels with IVIG and RTX using two-way ANOVA

	Sum of squares	df	η^2^	*F* ratio	*p* Value
IVIG	8.1	1	0.15	1.06	0.31
RTX	43.5	1	0.80	5.68	0.02
IVIG*RTX	2.5	1	0.05	0.32	0.58

### COVID-19 infections in IVIG vs non-IVIG group

COVID-19 infection until May 2022 was assessed, prior to the widespread of the Omicron variant. None of the patients in the IVIG group had COVID-19 infection before or after their vaccinations, while there were three cases in the non-IVIG group (one prior to vaccination and two patients after vaccination).

### Pre-vaccination samples

In order to control for false positivity, we also tested pre-vaccination plasma which was available in 30 patients (20 in the IVIG and 10 in the non-IVIG group). COVID ab levels were negative in all pre-vaccination samples including the seven samples collected after the pandemic onset (March 2020) which included four patients receiving IVIG.

## Discussion

To our knowledge, this is the first study to report COVID ab levels in patients receiving regular IVIG infusions. Higher monthly doses of IVIG in our IIM patients were significantly associated with higher levels of circulating COVID ab. Interestingly, the association of IVIG dose with COVID ab levels was strongest in patients receiving concomitant RTX therapy, a treatment shown to reduce COVID ab levels [11, 12] and associate with worse outcomes [13]. Comparison of IVIG and non-IVIG treatment groups of IIM patients did not reveal differences in COVID ab levels.

The possibility of passive transfer of antibodies to SARS-CoV2 was investigated early in the pandemic in studies of convalescent plasma. Kinetics of SARS-CoV-2 antibodies in high antibody titer convalescent plasma recipients showed short-term increase in anti-RBD and anti-spike antibodies [14–18]. However, antibody levels after 7–14 days and levels of other SARS-CoV2 antibodies were variable. In the current study, 67% of patients in the IVIG group were receiving infusions every 7 or 14 days, and results failed to show higher COVID ab levels compared to the non-IVIG group. Overall, these data show substantial variability in COVID ab levels after infusion with known COVID ab containing blood products.

Reports of IVIG products showing increasing levels of COVID ab as COVID-19 infection became widespread [2] and of IVIG preparations made from even pre-pandemic donors exhibiting both natural autoantibodies and cross-reactive antibodies to SARS-CoV2 [19] raising the question of whether IVIG products can lead to passive transfer of protective antibodies to its recipients. Among our IIM patients receiving IVIG, higher total monthly dose of IVIG was associated with higher COVID ab levels. The strongest associations of IVIG dose with COVID ab levels were noted in IIM patients concomitantly receiving RTX, which is intriguing given these patients are considered “high risk” with the suppressive effects of RTX on antibody production. RTX was significantly associated with lower COVID ab titers in our cohort, which is in agreement with other COVID-19 vaccine studies which have consistently demonstrated that patients treated with RTX have diminished serologic response [11, 12], one study reporting a 36-fold reduction in antibody levels [11].

COVID-19 vaccinations have substantially altered the course of the pandemic [1]. However, post-vaccination COVID ab titers are lower in patients with autoimmune diseases compared to the general population, and IIM patients have markedly lower post-vaccination seropositivity rates and ab levels compared to patients with rheumatoid arthritis (RA), systemic lupus erythematosus (SLE), psoriatic arthritis (PsA), and spondyloarthritis (AxSpA) [seropositivity 37% (IIM) vs 99% (AxSpA) vs 97% (PsA) vs 82% (RA) vs 92% (SLE)] [20]. These data highlight the importance of studying factors, which may influence COVID ab levels in IIM patients.

Our data show a clear correlation between the number of vaccination doses and higher COVID ab titers, suggesting that additional doses of mRNA vaccines provide further protective benefit as seen in the general population [21]. Other vaccine-related factors including mRNA vaccine type and timing from blood draw were not associated with COVID ab titers in our IIM cohort.

Medications that have been suggested to lower post-vaccination antibody levels such as prednisone and mycophenolate, [11, 22–24] were not associated with lower antibody levels in our cohort. One explanation for this may be that IIM patients have a lower immunogenic response overall to mRNA vaccines compared to other autoimmune diseases as demonstrated by Furer [20].

IVIG did not improve clinical outcomes in randomized trials of patients hospitalized with COVID-19 [7, 25]. However, the effect of IVIG in earlier COVID-19 disease stages and non-hospitalized patients is yet to be investigated. Certain patients groups, with preexisting conditions and at early stages of disease, showed benefit from convalescent plasma [8] even though randomized controlled trials failed to show clinical benefit [18]. In the current work, COVID infections occurred in three patients in the non-IVIG group while none occurred in the IVIG group patients before the spread of the omicron variant.

Our study has limitations. First, our study includes a small number of IIM patients from a single center. While the current sample size adequately demonstrated expected significant associations (number of vaccine doses and use of RTX), it was limited in performing robust multivariate analyses. Second, we analyzed anti-RBD IgG, while previous studies have also used IgM, IgA, antibodies against the complete spike protein, nucleocapsid protein, nucleocapsid-RNA binding domain and neutralizing assays [14, 15, 20]. However, the anti-RBD IgG levels strongly correlate with neutralizing antibody responses and are considered an acceptable surrogate of neutralization potency [26].

In conclusion, while the use of IVIG did not associate with higher COVID ab levels in comparison with IIM patients treated with IVIG as compared to an IIM control group not receiving IVIG, higher doses of IVIG did associate with higher circulating levels of COVID ab in patients receiving IVIG, particularly in patients concomitantly receiving RTX. Our findings suggest that IIM patients, particularly those at increased risk of COVID infection and worse COVID outcomes from RTX therapy, may have some protective benefits with higher COVID ab levels when on concurrent IVIG treatment. Further work is warranted to evaluate COVID infection outcomes in IIM patients receiving IVIG.

## Data Availability

Data from this work will be made available to investigators at academic institutions for noncommercial research upon request.
